# Functionalized Nanoparticles in Prevention and Targeted Therapy of Viral Diseases With Neurotropism Properties, Special Insight on COVID-19

**DOI:** 10.3389/fmicb.2021.767104

**Published:** 2021-11-15

**Authors:** Meishen Ren, Yin Wang, Yan Luo, Xueping Yao, Zexiao Yang, Pengfei Zhang, Wei Zhao, Dike Jiang

**Affiliations:** Animal Quarantine Laboratory, College of Veterinary Medicine, Sichuan Agricultural University, Chengdu, China

**Keywords:** neurotropic viruses, SARS-CoV-2, functionalized nanoparticles, drug delivery, prevention, targeted therapy

## Abstract

Neurotropic viruses have neural-invasive and neurovirulent properties to damage the central nervous system (CNS), leading to humans’ fatal symptoms. Neurotropic viruses comprise a lot of viruses, such as Zika virus (ZIKV), herpes simplex virus (HSV), rabies virus (RABV), and severe acute respiratory syndrome coronavirus type 2 (SARS-CoV-2). Effective therapy is needed to prevent infection by these viruses *in vivo* and *in vitro*. However, the blood-brain barrier (BBB) usually prevents macromolecules from entering the CNS, which challenges the usage of the traditional probes, antiviral drugs, or neutralizing antibodies in the CNS. Functionalized nanoparticles (NPs) have been increasingly reported in the targeted therapy of neurotropic viruses due to their sensitivity and targeting characteristics. Therefore, the present review outlines efficient functionalized NPs to further understand the recent trends, challenges, and prospects of these materials.

## Introduction

In human viral diseases, a large number of viruses have been reported in neurological diseases to invade the central nervous system (CNS) and cause severe damage to neuron cells, astrocytes, or microglia, leading to serious cerebral edema, encephalitis, and myelitis (Leung et al., 2007; Lafon, 2011; Lekgwara and Kelly, 2020; Masmejan et al., 2020). Common neurotropic viruses include Zika virus (ZIKV), herpes simplex virus (HSV), rabies virus (RABV), and recently reported SARS-CoV-2 (Ludlow et al., 2016; Alimonti et al., 2018; Tavčar et al., 2021). These viruses seriously endanger human life worldwide (Saiz et al., 2016; Fooks et al., 2017; Huang et al., 2020; Lee et al., 2020).

Neurotropic viruses are extremely pathogenic and have a high mortality rate. Especially for the patient infected by the RABV, there is almost no chance for disease recovery once the viral particle enters the CNS and starts replication (Leung et al., 2007). ZIKV was reported to be related to neurodevelopmental disorders in newborn children. Most of them suffered from microcephaly (van de Beek and Brouwer, 2017). In subsequent studies, the causal relationship between ZIKV infection and neurodevelopmental disorders has been further confirmed (Garcez et al., 2016; Baud et al., 2017). HSV is a typical neurotropic virus causing severe infection in the CNS, with the clinical symptoms of headache, fever, seizures, and cognitive impairment (Yordy et al., 2012). Since December 2019, SARS-CoV-2 infection broke out in Wuhan, China. At that time, viral genome sequencing and virus isolation were performed by Chinese scientists in January 2020 (Li et al., 2020; Zhou et al., 2020; Zhu et al., 2020). In a previous study, mammalian coronaviruses can infect the CNS, causing nerve cell damage (Hirano et al., 2004). Recent studies reported evidence for direct CNS invasion to confirmed that the SARS-CoV-2 exhibits neurotropism, leading to headache, loss of smell, confusion, and disabling stroke (Lekgwara and Kelly, 2020; Virhammar et al., 2020). In addition, the SARS-CoV-2 was found to infect hiPSC-derived neurons, astrocytes, and brain organoids, with astrocytes exhibiting a more severe response to viral infection (Iadecola et al., 2020; Wang et al., 2021b). So far, SARS-CoV-2 has caused severe damage to the safety of human life all over the world, leading to over 4 million deaths and 200 million confirmed cases (WHO 2021). The severe damage of SARS-CoV-2 to the safety of human life also caused social and economic crises (Meyer et al., 2021).

It takes a long time for human society to fight against emerging viral infectious diseases. With the development of nanotechnology, NPs-based viral vaccines have exhibited more design strategies and construction methods. The limitations of traditional viral vaccines, such as antigen stability, cell delivery, and targeted delivery, may be overcome by nanotechnology. Rapid and effective treatment of neurotropic virus infection is critical for human health. But it will be late for antiviral therapy using common drugs since the damage of neuronal cells is generally irreversible when neurotropic viruses replicate in large numbers in the brain (Wouk et al., 2021). For a desirable targeted therapy, the antiviral compound should not only be delivered to a specific tissue but should be accurately delivered to the virus-infected cells. In this case, antiviral agents can inhibit the virus in different ways, such as blocking the binding of the virus to the receptor on the cell membrane, inhibiting the replication of the virus in the cytoplasm, and inhibiting viral budding (Li et al., 2016; Park et al., 2016; Chen et al., 2021). Thus, targeted delivery of infected cells plays an essential role in promoting the therapeutic effect of antiviral drugs. On the other hand, the blood-brain barrier usually prevents drugs from entering the brain (Zhou et al., 2021). The drugs can be metabolized and lose their antiviral effect after blood circulation *in vivo*.

Given the challenges in the prevention and treatment of viral infection, it is vital to develop novel strategies for improving the efficacy of NPs-based vaccines and antiviral therapy. Thus, the principal aim of this review was to summarize the different functionalized NPs that were utilized for the infection of neurotropic viruses *in vitro* and *in vivo*. This review hopes to provide a fundamental understanding in designing the targeted nanoparticles for prevention and antiviral therapy of these diseases with neurotropism properties, especially COVID-19.

## Conventional Prevention and Treatment of Neurotropic Viral Infection

Common components for traditional prevention and treatment of neurotropic viruses include vaccines and antiviral drugs to promote the host antiviral innate and adaptive immunity for viral clearance (Vere Hodge and Field, 2013; Kamiyama et al., 2017; Smreczak et al., 2019).

According to previous studies, vaccines of neurotropic viruses have been extensively developed, including DNA vaccines, purified inactivated virus vaccines, mRNA vaccines, and viral vector vaccines (Johnston et al., 2016; Li et al., 2017; Tebas et al., 2017). Vaccines have a good therapeutic effect on most viral diseases because the viral immunogenic protein in the vaccine can stimulate the host to induce neutralizing antibodies to eliminate the virus. However, vaccination still has some limitations and challenges in conventional treatment. RABV vaccine has been studied for many years, and it has also played a critical role in the post-exposure prophylaxis (PEP; Conzelmann et al., 1990; Kessels et al., 2019). However, patients who received the neural tissue anti-rabies vaccine may have side effects in individual clinical cases, such as Guillain-Barre syndrome (Wajih Ullah et al., 2018). For the vaccine development of ZIKV, phase III clinical efficacy trials may be challenging to perform with the recent decrease of ZIKV transmission, although promising data were reported in some animal and phase I clinical trials (Abbink et al., 2018). From 2019 to 2021, the widespread of the virus has been controlled to some extent after humans were previously vaccinated with inactivated vaccines, recombinant spike protein expression vaccines, or mRNA vaccines all over the world (Amanat and Krammer, 2020). However, the emerging B.1.617.2 (Delta) variant might escape the neutralizing antibodies generated by vaccination or previous infection with SARS-CoV-2 (Planas et al., 2021). The vaccine effectiveness against the delta variant was found to exhibit modest differences compared to the alpha variant after two doses of vaccination (Lopez Bernal et al., 2021).

Antiviral compounds, such as ribavirin, remdesivir, and favipiravir, are broad-spectrum antiviral drugs that effectively inhibit viral replication through RNA-dependent RNA polymerase (RdRp; Vogt et al., 1987; Wang et al., 2020b; Kokic et al., 2021). These small molecule compounds were widely researched in antiviral therapy of different neurotropic viruses.

Favipiravir, namely, T-705 (6-fluoro-3-hydroxy-2-pyrazine carboxamide), is a selective and potential inhibitor for a broad spectrum of RNA viruses (Furuta et al., 2017). For example, this compound inhibited the viral replication of ZIKV in Vero cells at an EC_50_ of approximately 3.5μg/ml and was effective against RABV in murine neuroblastoma Neuro-2a cells with an EC_50_ of about 5.1–7.0μg/ml (Yamada et al., 2016; Zmurko et al., 2016). Favipiravir is an adenosine analog and has been recently reported as a potential antiviral agent for inhibiting SARS-CoV-2 at an EC_50_ of approximately 61.88μm/l in the Vero E6 cell model (Wang et al., 2020a). Ribavirin, a guanine nucleotide analog, was found to be effective for the viral inhibition of ZIKV and RABV replication *in vitro* (Kamiyama et al., 2017; Anindita et al., 2018). However, the application of ribavirin against SARS-CoV-2 is limited under the concentration of 100μm/l, according to recent studies (Choy et al., 2020). Its antiviral efficacy to inhibit viral replication needs high-dose and combination treatment, let alone its hematologic toxicity (Stockman et al., 2006).

For *in vivo* treatment, it was reported that favipiravir could reduce the peak median viral load of ZIKV by about 3 logs when the patients received a dose of 150mg/kg twice a day (Best et al., 2017). But the *in vivo* activity of T-705 against RABV is limited since it just delayed the onset of clinical symptoms when the virus was inoculated intramuscularly at 10^6.8^ TCID_50_/ml in a mouse model (Banyard et al., 2017). In clinical treatment, it is necessary to give a hefty dose of antiviral drugs to achieve the antiviral effects. On the other hand, side effects usually limit the *in vivo* therapy, such as hemolytic anemia, teratogen, and contraindicated in pregnancy caused by ribavirin (Stockman et al., 2006; Altınbas et al., 2020). For the antiviral treatment of COVID-19, although antiviral agents can inhibit the virus *in vitro*, the efficacy of treatment is still limited *in vivo*.

## Prevention and Antiviral Treatment By Functionalized Nps

**Figure 1:**
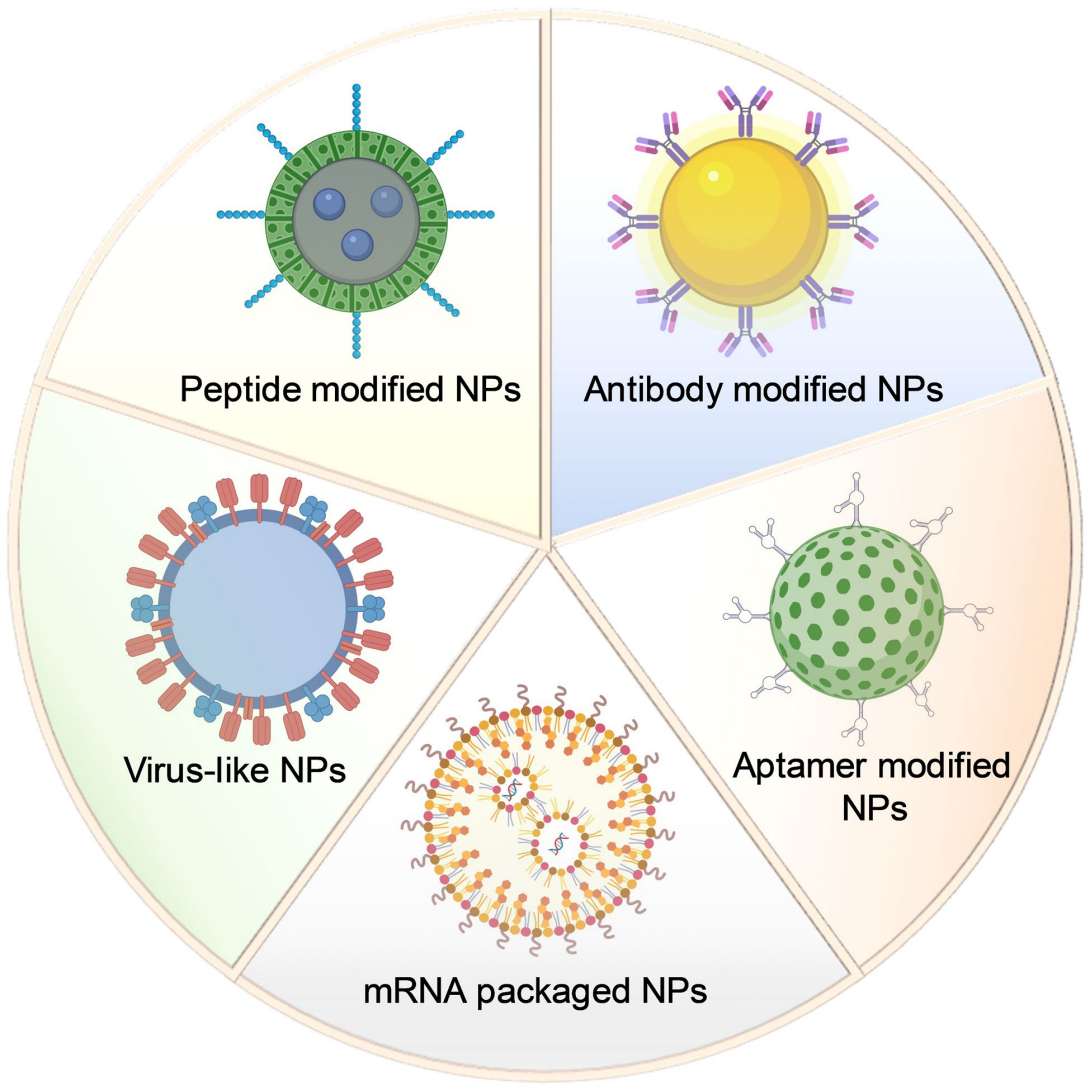
Schematic design of different types of nanoparticles in antiviral targeted therapy research.

After years of research, various types of functionalized NPs have been developed and validated in antiviral studies of different neurotropic diseases. For targeted treatment, antibodies, peptides, nucleic acid fragments, and antigenic components are usually conjugated with NPs to ensure the targeting of viral antigens or viral receptors (Figure 1). In addition, targeted delivery of drug nanocarriers is another crucial part of antiviral therapy, while functionalized NPs can achieve more efficient drug delivery compared with administration with conventional drugs alone.

### Peptide-Functionalized NPs

#### Peptide-NPs Mediated Prevention of Viral Infection

Peptide vaccines are widely used in anti-virus, anti-tumor, anti-bacterial, and anti-parasitic infection research. Peptide vaccines also have the advantages of low price, safety, strong specificity, and easy storage. However, its poor immunogenicity, insufficient efficacy, and short half-life also limit the application of traditional peptide vaccines.

The development of NPs vaccines is a crucial response against viral diseases. The rationally designed NPs vaccines can lead to improved immunogenicity of peptides since the peptides can be enriched on the nanosized nanoparticles to be abundantly displayed. Through different design strategies, peptide nanoparticles can enhance the biological stability of the peptide itself. Peptides derived from different viral antigens can also be used for the functionalization of NPs to trigger a strong immune response. For HSV infection, the virus spread by the cell-to-cell contact promotes the viral escape from the host immune response. Thus, a Toll-like receptor (TLR) 9 ligand CpG^m^ conjugated biodegradable calcium phosphate (CaP) NPs was coated with a neutralizing peptide to mimic a conformational epitope on HSV-1 gB to induce antibodies for inhibiting lethal infection *in vivo* (Kopp et al., 2019). Another AuNP was modified with a peptide derived from the B-cell epitope (S461-493) on the S protein of SARS-CoV-2, which exhibited a superior immune response compared to the soluble peptide (Farfán-Castro et al., 2021).

From the perspective of the compatibility of multiple peptides, the design of NPs also has more functional feasibility. In previous studies, a dendritic cells (DCs) binding peptide was expressed on the surface of recombinant viral particles by fusing to RABV glycoprotein protein (Zhang et al., 2018). This design can also be used in the construction of functional NPs. As a potential universal platform, NPs displaying different viral antigens could be combined to DCs binding peptides to enhance the activation of DCs and the generation of follicular helper T (T_FH_) cells and germinal center (GC) B cells. In addition, DCs binding peptides can be synthesized and modified in a controlled manner according to the dose ratio for functionalized NPs. Besides, the construction of recombinant chimeric virus vaccines requires complicated screening of the insertion sites of DCs binding peptides, but NPs only require simple surface modification. Therefore, the design of peptide-NPs vaccines shows a more flexible possibility than traditional peptide vaccines.

#### Peptide-NPs Mediated Antiviral Therapy

Artificially synthesized peptides are often used for the functional modification of NPs. For viruses, the envelope structure plays an essential role in binding the virus to host cells. Therefore, destroying the integrity of the envelope structure can effectively inhibit virus entry. In antiviral research, some peptides have antiviral capabilities, such as a synthetic peptide, namely, AH, which was found to cause rupture and disintegration of enveloped viruses by transforming the envelope membrane into planar bilayer (Cho et al., 2009; Jackman et al., 2013). In later studies, this peptide has been reported to target viral envelopes of HIV, west nile virus (WNV), and dengue virus (DENV; Bobardt et al., 2008; Cheng et al., 2008; Hanson et al., 2016).

Another targeting peptide (gH625-644) conjugated at the termini of a poly(amide)-based dendrimer was derived from the HSV-1 glycoprotein H and has been found to interact with membrane to inhibit infection of both HSV-1 and HSV-2 (Tarallo et al., 2013). Similarly, LL-37 is a 37 amino acid peptide exhibiting antimicrobial and antiviral effects derived from the C-terminal of the human cationic antimicrobial protein (hCAP18; Lehrer and Ganz, 2002). This peptide was reported as a modified component on a composite nanoparticle-hydrogel corneal implant, showing antiviral activity against HSV-1 infection by blocking the viral binding to the cells (Lee et al., 2014). For ZIKV, a synthetic peptide derived from the stem region of viral envelope protein could interact with the viral surface antigen to destroy the integrity of the envelope structure. It was found to penetrate the placental barrier to inhibit ZIKV infection in both pregnant mice and fetuses (Yu et al., 2017). Another peptide derived from the stem of the Japanese encephalitis virus (JEV) envelope glycoprotein also exhibited the ability to block ZIKV infection (Chen et al., 2017).

For peptide-mediated target treatment of SARS-CoV-2 infection, the angiotensin-converting enzyme 2 (ACE2) cellular receptor and the receptor-binding domain (RBD) of spike protein (S protein) are significant targets for blocking the virus from binding to human cell receptors. Many peptides are screened and identified based on these two target molecules. In early research, some α-helix fragments on ACE2 were found to interact with the residue on RBD and a full cover of the RBD surface, which provides a theoretical basis for the subsequent research on peptide-functionalized NPs and peptide-based vaccines (Han and Král, 2020; Lim et al., 2021). As reported in a later study, a truncated ACE2 peptide was conjugated on the surface of gold nanoparticle (AuNP) and was found to bind with the SARS-CoV-2 RBD with a high affinity of 41nm, demonstrating a potential application in antiviral research against COVID-19 (Mesias et al., 2021).

**Figure 2:**
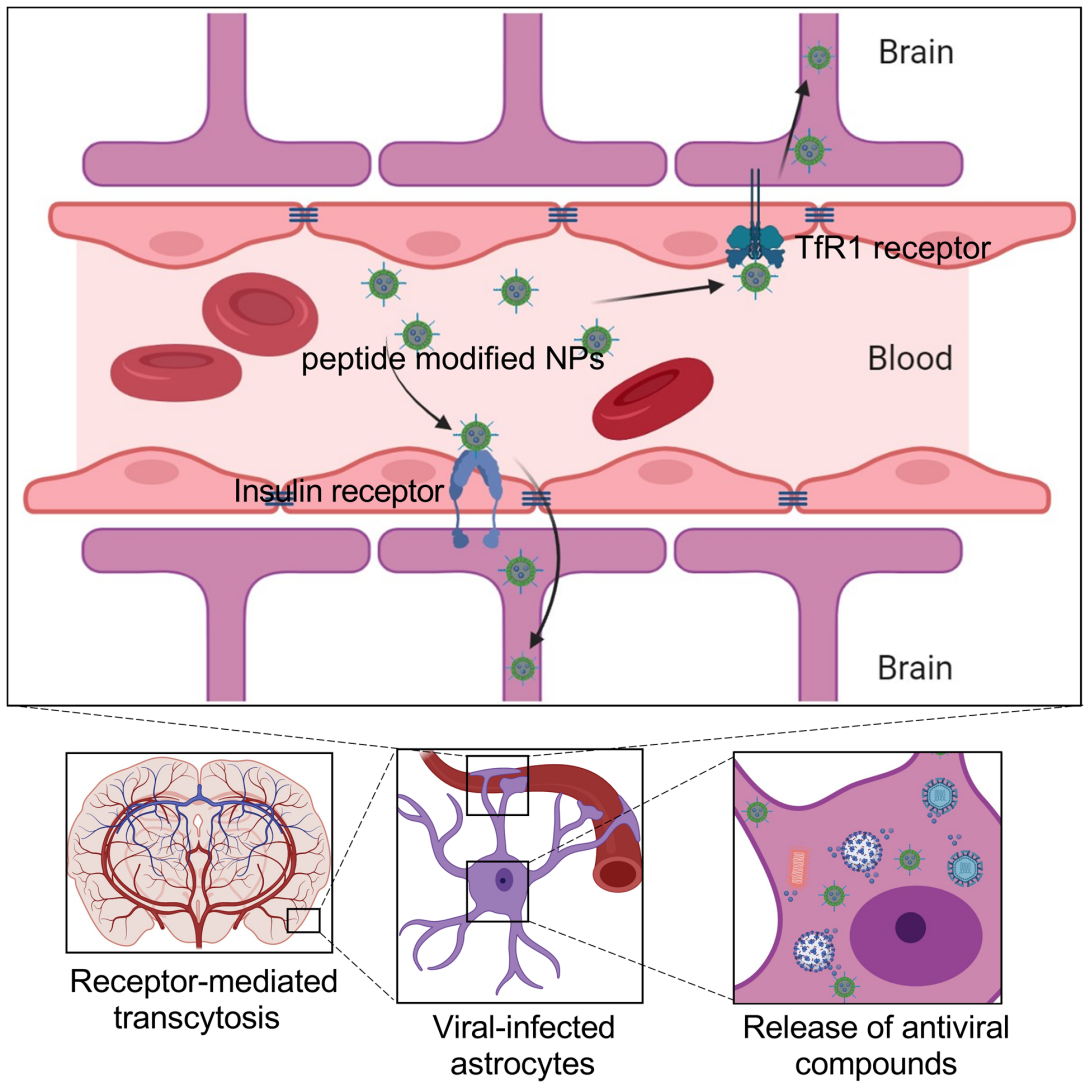
Schematic showing the brain delivery of NPs across the BBB, which can be administered *via* the intravenous route. NPs penetrate the BBB through receptor-mediated transcytosis by coupling peptides or antibodies that enable nanoparticles to bind to receptors on the surface of endothelial cells. Such NPs encapsulate the antiviral drugs and specifically release them in the infected cells.

The BBB mainly consists of the tight brain endothelium surrounded by the basal lamina and mediated by neurons, glia, and pericytes (Abbott et al., 2010). In the conventional treatment of the neurotropic virus, it is difficult for the antiviral compounds to pass through the BBB, let alone further inhibition of viruses in the CNS. Therefore, improving the CNS delivery efficiency is critical for the therapy of viral diseases with neurotropism properties. In recent research, the mechanisms of transport pathways of BBB shuttles include receptor-mediated transcytosis, carrier-mediated transport, transcellular passive diffusion, and adsorptive-mediated transcytosis. The receptor-mediated transcytosis is the main pathway for BBB crossing by peptides, including transferrin (TfR1), low-density lipoproteins (LDLRs), and insulin (Figure 2; Duffy and Pardridge, 1987; Dehouck et al., 1997; Qian et al., 2002). Especially TfR1 and LDLRs are highly expressed in the brain endothelium cells. Peptide-functionalized NPs are easier to enter or bind the neural cells to perform their antiviral function, such as drug and antigen release, neutralizing antibody binding, antiviral photothermal effect.

**Figure 3:**
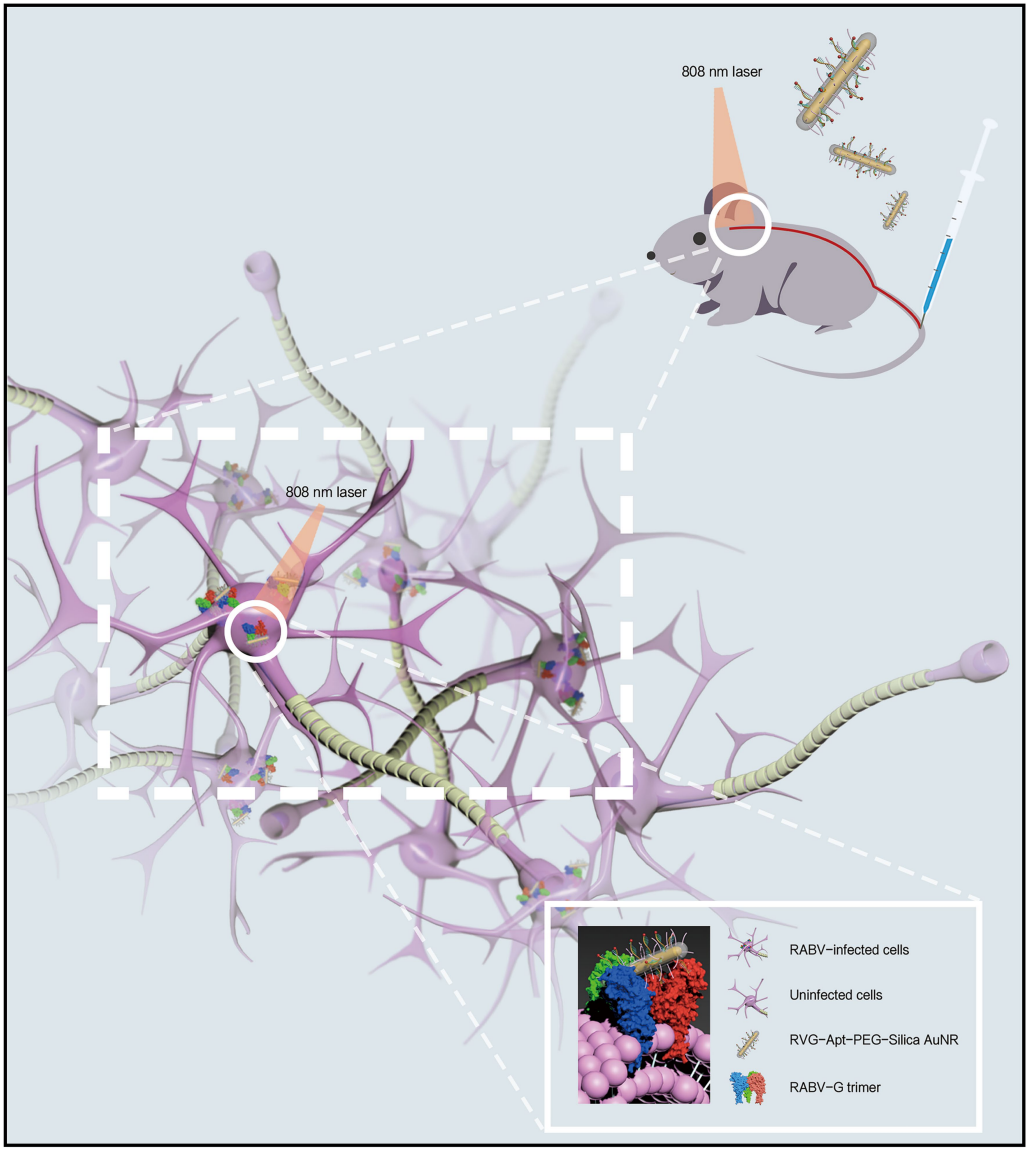
Schematic diagram of CNS delivery and viral-targeting photothermal therapy by a rabies virus glycopeptide (RVG) peptide-functionalized NPs.

For the treatment of neurotropic virus infection, promoting brain delivery efficiency is vital to improve the therapeutic effect. As a component, short peptides have been reported to enhance the CNS tropism of brain delivery NPs, for example, the RABV glycopeptide (RVG; Figure 3; Lee et al., 2017). This peptide contains 29 amino acids derived from RABV glycoprotein, promoting the viral transportation from the peripheral nerve to the CNS (Oswald et al., 2017). In terms of mechanism, the RVG peptide can bind the γ-aminobutyric acid receptor (GABA) and the nicotinic acetylcholine receptor (nAChR) to enter the peripheral nerve cells and CNS (Kumar et al., 2007; Liu et al., 2009). Thus, the RVG peptide has been applied in brain-targeted functionalized NPs in recent studies.

In a rabies treatment, an RVG and DNA aptamer conjugated gold nanorod (AuNR) has been found to display improved CNS tropism and targeting ability to viral glycoprotein (Ren et al., 2021). Specifically, by surface modification of RVG peptide, this AuNR was delivered into CNS post intravenous (i.v.) injection in a mouse model. The nAChR receptor is also expressed on neuronal cells and astrocytes (Lendvai and Vizi, 2008; Matta et al., 2021). The RVG functionalized AuNR can be accumulated at the cell surface receptor or enter the cells, promoting viral inhibition since RABV mainly infects these two cells in CNS (Piccinotti and Whelan, 2016; Tian et al., 2017). As is indicated in this study, the functionalized AuNR inhibits the viral infection by blocking the receptor site on the neuronal cells and astrocytes while targeting the membrane antigen on virions by a DNA aptamer to inactivate the virus by photothermal effect induced by AuNR and near-infrared spectrum (NIR) irradiation (Ren et al., 2021).

In addition to entering the CNS through the peripheral nerves, another strategy is to increase the permeability of the blood-brain barrier to enhance the BBB crossing efficiency. For example, a cell-penetrating peptide that derived from the nucleolar translocation signal peptide of the LIM Kinase 2 protein improved the BBB crossing efficiency of a dendrigraft poly-L-lysines (DGL)- and polyethyleneglycol (PEG)-based gene vector in a mouse model (Yao et al., 2015). An engineered brain-penetrating peptide, namely, AH-D, has also shown the ability to cross the BBB to reduce the viral loads in ZIKV-infected mouse brains and protected against the damage of BBB induced by ZIKV (Kamiyama et al., 2017).

### Antibody, Receptor, and Antigen Functionalized Nanoparticles for Prevention and Targeted Treatment

#### Prevention of Viral Infection by Antigen Functionalized NPs

NPs enriched with antibodies or receptor molecules can be used to directly bind and block viruses to inhibit the virus from infecting the host cells. Unlike directly blocking viral particles to target cells by antibodies or receptors, the viral glycoprotein can be enriched and displayed on the surface of the NPs to induce a strong immune response for antiviral prevention. Like traditional protein subunit vaccines, the antigenic proteins displayed on the NPs have good safety and easy to obtain. As a universal vaccine technology platform, viral antigen protein enriched NPs can be used as a promising vaccine candidate for preventing infection of different viruses.

In a previous study, a synthetic virus-like particle (VLP), called RBD-SpyVLP, was abundantly modified with the spike glycoprotein RBD. This RBD-SpyVLP has induced a robust neutralizing antibody immune response in mice, even when administered at a low dosage. Interestingly, the antibody response is negligible in the mice vaccinated with an equal dose (0.1 or 0.5μg) of purified RBD. In previous studies, the purified RBD can induce neutralizing antibodies *in vivo* under higher dosage and more frequent injections (Zhang et al., 2016; Yang et al., 2020). However, the antibody response is significantly strong when the RBD is abundantly displayed on the RBD-SpyVLP. In virus neutralization experiments, antibodies elicited by this VLP were able to inhibit the pseudovirus or wild-type SARS-CoV-2. In addition, this RBD-SpyVLP is thermally stable and retains immunogenicity after being lyophilized, which promotes the popularization of vaccines and cold-chain transportation (Tan et al., 2021). As a promising vaccine platform to facilitate conjugation with other antigens, more than one RBD variant can be co-displayed on the NPs to provide cross-protection for different variants of SARS-CoV-2, such as Alpha variant and Delta variant.

Self-assembling protein vaccine is another method to display the glycoprotein on the surface of NPs to develop a robust immune response for antiviral therapy. In the treatment of ZIKV, a self-assembling nano-vaccine has been established after supplying flanking sequences at both the N- and C-terminal of nonstructural protein 1 (NS1) by genetic engineering. According to their results, this vaccine formed a 3-dimensional structure by self-assembling with enhanced immunogenicity and improved longevity in comparison with unmodified NS1 in vaccinated mice (Favaro et al., 2021).

#### Antibody and Receptor-Modified NPs for Antiviral Therapy

Monoclonal antibodies and polyclonal antibodies can be easily obtained through conventional antibody preparation technology. For most virions, the immunogenic protein is located on the surface of the virus particle. Specific targeting by antibodies to bind structural protein on the surface of the viral particles can inhibit viral surface antigen-mediated cell receptor binding to prevent virus invasion into host cells (Magnani et al., 2017). As a result, the viral life cycle is interrupted since the virus cannot enter the host cells to complete genome replication and progeny virus assembly.

In a recent study, liposomal-based Nanotrap surfaces were functionalized with SARS-CoV-2 neutralizing antibody and phagocytosis-specific phosphatidylserines (Chen et al., 2021). This Nanotrap has been found to completely inhibit SARS-CoV-2 infection by blocking the binding of S protein to ACE2 of host cells. It significantly inhibited the infection by a pseudotyped and authentic SARS-CoV-2 in an *ex vivo* lung perfusion system (Chen et al., 2021). Moreover, this Nanotrap promotes the phagocytosis of macrophages without being infected themselves after surface modification of the phagocyte-specific phosphatidylserine ligands. The ACE2 protein is another ideal bait to trap S protein to block SARS-CoV-2 infection. In a membrane extrusion ACE2-NPs derived from human embryonic kidney-293T cells, ACE2 was abundantly expressed on the surface at the dose concentration of 265.1ng/mg. As a result, the ACE2-NPs significantly suppressed the cell entry of pseudotyped SARS-CoV-2 and inhibited viral infection *in vitro* and *in vivo* (Wang et al., 2021a). In addition, the ACE2-NPs were found to reduce the apoptosis caused by promoting the expression of optic atrophy 1 (OPA1) protein to inhibit the release of cytochrome C (Wang et al., 2021a).

### Prevention and Antiviral Therapy by Nucleotide Nanoparticles

#### Prevention of Viral Infection by Nucleotide-NPs

Artificially designed and synthesized DNA or RNA is usually one of the components of NPs to obtain specific properties. Nucleotide vaccine is a promising technology to induce an immune response to prevent virus infection. However, the traditional mRNA vaccines often face the challenges of delivery, cellular uptake, and degradation *in vivo*. The naked mRNA vaccine is degraded and eliminated by RNases and endonucleases in the serum when administered intravenously (IV). In addition, the viral mRNAs can activate the pattern-recognition receptors (PPP) as a pathogen-associated molecular pattern (PAMP). For example, the viral single-stranded RNA (ssRNA) can be recognized by TLR to trigger innate immune activation, causing inflammation and immunogenicity (Diebold, 2008). Besides, mRNA vaccines need to enter cells to express antigen proteins to induce adaptive immunity. The naked mRNA also exhibited poor cellular uptake due to their negatively charged surface potential. The lipid nanoparticles (LNPs) can encapsulate and deliver nucleic acid vaccines to particular tissues or cells to express antigen proteins to cause host immune response (Reichmuth et al., 2016). In a ZIKV antiviral study, a replicating viral RNA of ZIKV antigens has been combined with a highly stable nanostructured lipid vector, demonstrating a single dose as low as 10ng could completely protect an acute virus challenge in a mouse model (Erasmus et al., 2018). This strategy was found to reduce the duration of viremia of ZIKV with a single dose as low as 10ng. In addition, intracellular delivery by the NPs also reduced the reactogenicity through less formulation per dose. Another mRNA vaccine expressing ZIKV prM and E proteins encapsulated in lipid NPs has been reported to generate robust neutralizing antibody response in female mice and restrict *in utero* transmission of ZIKV to the fetus after being pregnant (Richner et al., 2017).

#### Antiviral Therapy by Nucleotide-NPs

Aptamer usually consists of single-strand DNAs or RNAs, exhibiting high affinity to target molecules, such as receptors on the surface of living cells, viral antigen proteins, and chemical compounds (Zhou and Rossi, 2017). Due to the flexibility to form complementary secondary structures, aptamers can recognize specific molecules through three-dimensional interactions using unique tertiary structures. Like antibodies binding to the antigens, the selected aptamers can generate aptamer-target complexes by different types of interaction, such as hydrogen bonding, hydrophobic interaction, and van der Waals forces (Gelinas et al., 2016). In the past decades, aptamer-based NPs for antiviral therapeutics have been widely studied. Although the development in clinical research is not as good as that of antibodies, aptamer-NPs are advantaged in their flexibility to recognize those unique binding sites which may not be accessible to antibodies. Thus, the therapeutic nucleic acid aptamer-NPs also have great potential in the field of antiviral researches.

Similar to antibodies, the ectodomains of viral glycoprotein are usually an ideal target for the specific selection of DNA/RNA aptamer in some antiviral research. For example, two RNA aptamers have been selected and isolated to specifically interact with the gD protein of HSV-1 with robust affinity. According to their results in plaque assays, the aptamer-NPs inhibited HSV-1 in a dose-dependent manner with the 50% inhibitory concentration (IC_50_) of 0.8μm (Gopinath et al., 2012). Another 45-nt-long DNA aptamer has also been selected and showed high affinity to HSV-1 gD protein, with an affinity constant of 50nm. This study has demonstrated that this 45-nt-long DNA aptamer significantly restricts the viral entry and replication both *in vitro* and *in vivo* (Yadavalli et al., 2017). According to the above two studies, the glycoprotein should be a suitable target for aptamer design to locally reduce the viral spread of infection. The spike protein of SARS-CoV-2 is a necessary immunogenic antigen binding to the ACE2 receptor on the host cells. In recent studies, specific aptamers have been isolated to interact with ACE2 to block SARS-CoV-2 infection. For example, a DNA aptamer, namely, cb-CoV2-6C3, has been confirmed stable in serum solution for at least 12h with a strong affinity (K_d_ of 0.13nm) to target ACE2 for viral inhibition at an IC_50_ of 0.42nm. Moreover, this aptamer cb-CoV2-6C3 can be sustained under room temperature for more than 14days (Sun et al., 2021). At the molecular level, the aptamer mainly targets the amino acid sites Phe 486 and Gln 474 at the C-terminal of the RBD domain of S protein. Another DNA aptamer, called SP6, has also been demonstrated binding to S protein with the potential to inhibit SARS-CoV-2 infection. However, the inhibition mode of aptamer SP6 distinguishes it from that of antibodies targeting the RBD domain of S protein. Since, according to this study, the inhibitory of SP6 does not cause by interfering with the binding of SP6 aptamer to RBD on the S protein. The authors indicated that the inhibitory molecular mechanism needs to be further studied to provide knowledge of S protein fusion to the host cell membranes (Schmitz et al., 2021).

The selected aptamers are usually smaller than the purified antibodies. Compared with antibodies, more aptamer molecules can be accommodated in an equal volume around the target molecule, promoting the local concentration of administrated aptamers to obtain better antiviral efficacy. In addition, the smaller size also provides aptamers a potential to be directly delivered into CNS or the respiratory system, instead of being obstructed by the blood-gas barrier or blood-brain barrier. Notably, the antiviral treatments of COVID-19 by traditional unmodified neutralization antibodies have been reported facing potential problems of antibody-dependent enhancement (ADE; Arvin et al., 2020). But this potential risk has not yet been found in the researches of aptamer antiviral therapeutics, especially for SARS-CoV-2 infection.

### Other Applications of Inorganic and Modified Compound Nanoparticles

The direct antiviral researches by inorganic NPs are gradually increasing. For example, gold- and silver-based NPs have received increasing attention due to their anti-bacterial and antiviral properties. A new gold nanoparticle family was found to inhibit the infection of HSV-1 in a neural-derived cell model (Rodriguez-Izquierdo et al., 2020). Similarly, silver nanoparticles (Ag-NPs) were reported to inhibit the viral replication of HSV-2 when administered previous to virus infection (Hu et al., 2014).

The glycyrrhizic acid (GA), namely, glycyrrhizin, is a usual ingredient in the Chinese herb licorice which has been applied in antiviral research against various viruses and viral diseases, such as SARS-associated coronaviruses and viral hepatitis (Cinatl et al., 2003; Li et al., 2019). According to this property, a kind of highly biocompatible glycyrrhizic acid nanoparticles (GANPs) has been synthesized in antiviral research of SARS-CoV-2 infection *in vitro* and *in vivo*. In this study, the GANPs have exhibited no significant toxicity and improved biocompatibility. The anti-inflammatory effect has been found to relieve the excessive inflammation induced by SARS-CoV-2 since the GANPs could target the locations of severe inflammation by enhanced permeability and retention (EPR) effect in a mouse model (Zhao et al., 2021). Thus, the modified compound also can be a possible antiviral candidate against COVID-19 in future studies.

## Conclusions and Future Perspectives

The neurotropic viruses and their related diseases have caused an unprecedented economic crisis and a massive threat to life safety, such as ZIKV, HSV-1, HSV-2, RABV, and especially SARS-CoV-2. Since the initial outbreak of SARS-CoV-2 in December 2019, more than 4 million deaths and 200 million confirmed cases have been reported worldwide. Conventional antiviral agents, such as ribavirin, remdesivir, and favipiravir, have been widely studied for the treatment of infections induced by these viruses. But the drug targeting ability, permeability of blood-brain barrier and blood-gas barrier, *in vivo* stability, and pharmacokinetics are major challenges preventing the further application for antiviral therapy in clinical researches. If these technical difficulties cannot be overcome, clinical medications will have to be treated with high-dose and frequent administration, which will aggravate the cytotoxicity of the drug and damage the kidney and liver of the patient. Based on the above considerations, we reviewed various functionalized NPs in targeted therapy of viral diseases with neurotropism properties, especially COVID-19. The various morphology, diversity, and possibility to surface modification with different types of compounds have demonstrated that biomaterials have the potential in nanomedicine against these viral diseases.

For the construction of functionalized NPs, there are multiple ways to target different kinds of molecules. By modifying antibodies, peptides, and aptamers on the surface of NPs, the NPs can strongly and specifically recognize viral antigenic proteins, which provides a theoretical basis and technical support to enhance the specificity of traditional antiviral drugs. Further, nanoparticles with viral specificity can be designed to inhibit major steps of the viral life cycle. In the case of antibody-modified NPs, for example, glycoproteins on the surface of vesicular virions and viral antigenic proteins displayed on the surface of infected cells can directly interact with the antibody-modified NPs, thereby blocking viral particle binding to cellular receptors or inhibiting the assembly and budding of the virus within the infected cells.

**Figure 4:**
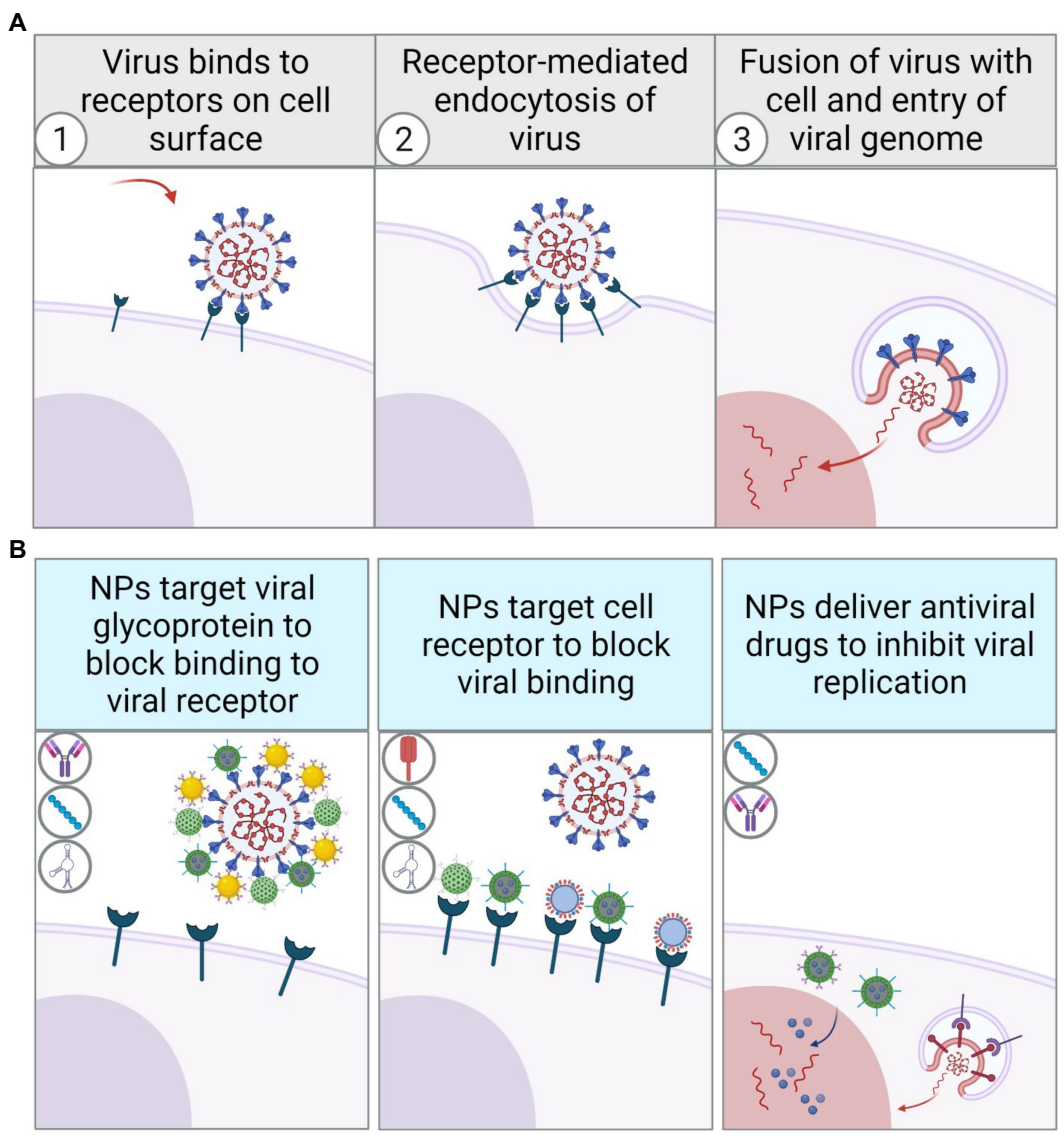
Schematic diagram of virus recognizing cell surface receptors to invade and replicate in host cells through receptor-mediated endocytosis **(A)**. Schematic diagram of viral inhibition by functionalized NPs with different surface modifications **(B)**.

No matter what kind of component is used to modify the functionalized NPs, the fundamental mechanism is to target viral antigens or cell surface receptors through their surface molecules to block viral particles from binding to target cells. In such a process, different types of functionalized NPs have specific features, advantages, and limitations. When designing therapeutically targeted nanoparticles, the applicability of various components in *in vivo* experiments should be fully considered. Therefore, these targeted NPs have also been statistically compared to better understand their characteristics in the further application (Table 1). Besides, NPs can also inhibit viral genome replication by facilitating drug delivery in viral-infected cells (Figure 4). NPs with viral specificity also promote cellular transcytosis, making it easier for nanoparticles to enter the infected cells and thus perform different functions. For instance, after the drug-encapsulated nanoparticles enter the infected cells, the release of antiviral drugs can inhibit viral replication more effectively than direct administration. On the one hand, some viruses escape the innate immune response intracellularly to promote viral replication, for example, by inhibiting the activation of inflammation-related signaling pathways. In such cases, viral-specific NPs encapsulated with TLR agonists or reactive oxygen species (ROS) are designed to artificially enhance innate immune response and inhibit immune escape in the early stages of viral infection, thereby promoting dendritic cells (DCs) activation, antigen presentation, and adaptive immune response. On the other hand, some viral infections also cause intense cellular inflammation. Viral-specific NPs can suppress the inflammation in infected cells by encapsulating and releasing anti-inflammatory inhibitors or cytokines such as interleukins 4, 10, 13 (Opal and DePalo, 2000). Similarly, the antiviral therapy by photothermal effect stimulated by NIR spectroscopy also benefits from virus-targeted NPs. When NPs with photothermal properties are clustered explicitly on the surface of virus particles, NIR irradiation causes the nanoparticles to convert light energy into heat energy, which inactivates the adjacent virions.

**Table 1:** Summary of the functionalized NPs in targeted therapy of viral infections.

Components	Advantages and features	Application examples	Possible limitations	References
Peptide-NPs	Broad-spectrum targets different molecules. Easily synthesized. Small molecular weight to penetrate tissues	Peptide (AH)-NP can target viral envelopes of HIV, WNV, DENV. SARS-CoV-2 related peptide-NPs can target the ACE2 receptor and RBD domain of viral antigen	Recognize a single epitope and cannot completely cover all epitopes on one molecule. Its sequence fragments need to be strictly screened	Bobardt et al., 2008; Ren et al., 2021; Hanson et al., 2016; Han and Král, 2020; Lim et al., 2021
Antibody-NPs	High affinity to viral antigens or host cell surface receptors. The neutralizing antibody itself can neutralize the virus to promote the therapy. Cover multiple epitopes on one molecule	Capture and block infection of SARS-CoV-2 while promoting the phagocytosis of macrophages by a liposomal-based Nanotrap NP	Due to the relatively large molecular weight, it is more difficult for antibody-NPs to penetrate tissues in drug delivery systems	Chen et al., 2021
Receptor-NPs	High affinity to viral antigens to block virus binding to the host cell receptors	Capture and block infection of SARS-CoV-2 by an ACE2-NP *in vitro* and *in vivo*	The tertiary receptor structure needs to be considered before constructing receptor-NPs to ensure its epitopes are not blocked	Wang et al., 2021a
Antigen-NPs	Competitively binds virus target cell receptors to inhibit virus invasion while stimulating the host immune response to eliminate the virus further	An RBD-SpyVLP abundantly displayed the RBD domain of SARS-CoV-2 to induce a robust neutralizing antibody immune response	In acute infections, competitive inhibition is not as effective as the direct neutralization of antibodies. The immune response may occur later than the infection treatment window	Tan et al., 2021
Nucleotide-NPs	Broad-spectrum targets different molecules. Easily synthesized. Multifunctional sensors based on nucleotide aptamers can be constructed, such as virus targeting and visualization	DNA aptamer (cb-CoV2-6C3) targets the ACE2 for SARS-CoV-2 inhibition at an IC_50_ of 0.42nm	The naked nucleotide components are vulnerable to degradation and charge effect, which need stable NPs to deliver	Ren et al., 2021; Gopinath et al., 2012; Yadavalli et al., 2017; Sun et al., 2021

The molecular weight, as well as the unit volume of the antibodies, are relatively larger compared to the general NPs. It may limit the number of NPs aggregated near the antigen proteins or targeted molecules. In this regard, nucleic acid aptamers and specific peptides can be used as alternatives to antibodies for the modification of NPs due to their lower molecular weight and space size. However, they may do not cover the entire site of antigenic epitopes, so it is an optional strategy to use peptides or aptamers with different antigenic epitopes in combination if necessary. Moreover, smaller molecules like peptides can be used to facilitate the functionalized NPs crossing the BBB due to their ability to specifically target nerve cells or BBB endothelial cell surface receptors. Besides, the covalent coupling of antibodies to NPs may exist in a variety of forms. It is possible that NPs have the potential to bind in the variable regions of the antibody molecule if simply coupling the amino and carboxyl of both molecules. Such NPs cannot capture the target molecule, as they block the antigen recognition site on the antibody. For example, an optimal modification strategy may need to consider coupling NPs to the heavy chain constant region of the antibody.

So far, a large number of functionalized nanoparticles with different functions and properties have been studied and reported. Here, we focus on their targeting, delivery efficiency, and antiviral effects against several viral diseases with neurotropism properties, especially COVID-19. An in-depth understanding of the design and construction of functionalized nanoparticles can provide a dual perspective of biology and chemistry to examine and address the difficulties faced by conventional biology or pharmacology in antiviral treatment. However, despite the advantages of functional NPs, their metabolism, degradation, and *in vivo* non-specific adsorption are inherent problems. Generally speaking, the NPs-based therapy needs to consider biological safety *in vivo*. Excessive accumulation of NPs may cause cell damage and even affect the normal function of tissues and organs (Piscatelli et al., 2021). After the NPs are metabolized by blood circulation, it is common to accumulate in the liver to a certain extent. In severe cases, it will affect the kidneys (Zarska et al., 2018). Since the liver and kidney play a vital role in the circulation and metabolism of the body, the externally injected nanoparticles will inevitably accumulate in such internal organs, which need to be gradually excreted from the body during an extended period of recovery. However, complications and possible adverse reactions may occur simultaneously and cause unexpected disorders, such as gastrointestinal, blood, lymphatic, and nervous system disorders (Table 2). Therefore, in the future antiviral research of functionalized NPs, while paying attention to the antiviral efficacy, biological safety cannot be ignored as well. More efforts are needed to improve the metabolism of NPs *in vivo* and reduce potential complications and adverse events while maintaining sufficient curative efficacy.

**Table 2:** Examples of NPs inducing potential complications and adverse events in some clinical trials.

Examples in clinical trials.gov (Identifier No.)	Administration routes	General disorders	Musculoskeletal and connective tissue disorders	Gastrointestinal disorders	Nervous system disorders	Blood and lymphatic disorders	Skin and subcutaneous tissue disorders	Construction strategies
NCT00629499	Intravenously	Fatigue	NA	Hemorrhage, vomiting	Mental Status	Hemoglobin, neutrophils	Rash, alopecia	Nab-paclitaxel-containing adjuvant NPs
NCT01620190	Interventional therapy	Fatigue	NA	NA	Peripheral Neuropathy	Hypotension, Neutrophil count decreased, White blood cells decreased	NA	Paclitaxel albumin-stabilized NPs
NCT02009332	Intravesical	Malaise	Worsening of edema of extremities	Nausea	Headache	Anemia,	Mucositis	A sterile lyophilized powder of albumin-bound sirolimus NPs
NCT00748553	Interventional therapy	Chills, Edema, Fever	Arthralgia, Muscle Weakness	Constipation,Diarrhea, Dry mouth, Hemorrhoids	Dizziness, Dysgeusia	Anemia, Thrombocy-topenia	Alopecia	Hypomethylating agent azacitidine (Vidaza) with the NPs albumin-bound paclitaxel (Abraxane)

*All data in Table 2 were obtained from the database of the clinical trials.gov (https://clinicaltrials.gov/ct2/home) NA: not applicable*

## Author Contributions

MR designed and wrote this paper. All authors listed have made a substantial, direct and intellectual contribution to the work and approved it for publication.

## Funding

This work was funded by the Sichuan Province Science and Technology Planning Project (project 2020YJ0345).

## Conflict of Interest

The authors declare that the research was conducted in the absence of any commercial or financial relationships that could be construed as a potential conflict of interest.

## Publisher’s Note

All claims expressed in this article are solely those of the authors and do not necessarily represent those of their affiliated organizations, or those of the publisher, the editors and the reviewers. Any product that may be evaluated in this article, or claim that may be made by its manufacturer, is not guaranteed or endorsed by the publisher.
